# Fast-Track Crystallization of PB‑1 with Sorbitol-Based Nucleating Agent

**DOI:** 10.1021/acsomega.5c08056

**Published:** 2025-10-20

**Authors:** Jana Navratilova, Lenka Gajzlerova, Roman Cermak, Martina Polaskova

**Affiliations:** † Department of Polymer Engineering, Faculty of Technology, 48362 Tomas Bata University in Zlin, Vavreckova 5669, 760 01 Zlin, Czech Republic; ‡ The Centre of Polymer Systems, Tomas Bata University in Zlin, trida Tomase Bati 5678, 760 01 Zlin, Czech Republic

## Abstract

The work is focused on crystallization and phase transition of polybut-1-ene (PB-1) homopolymer and random copolymer in the presence of commonly used nucleating agent (NA) 1,3:2,4-bis­(3,4-dimethylbenzylidene) sorbitol, primarily designed for polypropylene. The crystallization and subsequent phase transition from kinetically preferred tetragonal form II into thermodynamically stable trigonal form I in defined times of aging at room temperature is studied. It was found that NA accelerates phase transition, particularly in homopolymer. In the copolymer, moreover, the addition of NA dramatically increases the crystallization temperature and narrows the crystallization peak as well as the melting peak, which corresponds to the formation of a more uniform structure. At the same time, the addition of NA increases the haze significantly, confirming the formation of a two-form structure. However, NA was found to be soluble in a low concentration (0.2 wt %) in PB-1 melt at a sufficiently high temperature of melting. The solubility then affects the crystallization behavior. Finally, it can be confirmed that the addition of commercial sorbitol-based NA has a significant effect on the crystallization and phase transition of both the PB-1 homopolymer and the copolymer.

## Graphical abstract

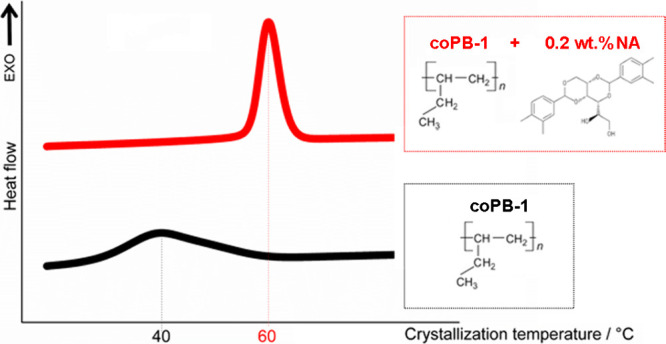

## 1 Introduction

The crystallization behavior of polymers is a key factor in determining their mechanical, optical, and thermal properties. Among polyolefins, polybut-1-ene (PB-1) stands out for its significant polymorphism, characterized by time-dependent phase transitions between different crystalline modifications. The initial crystallization of PB-1 usually leads to the kinetically preferred metastable form II, which has a tetragonal crystal structure with an 11/3 helical conformation. This form gradually and irreversibly changes to the thermodynamically stable form I, which has a trigonal crystal structure with a 3/1 helical conformation.^1–4^ The transition from form II to form I is accompanied by deformation of the crystal lattice, corresponding to lateral shrinkage of approximately 20% and normal elongation of approximately 12%.^5^ This transformation has a significant effect on the transparency, mechanical strength, and long-term stability of the material. The entire transformation process at room temperature takes days to weeks, which significantly increases production costs due to the need for long-term storage.^6–8^

The PB-1 phase transformation is a complex solid-to-solid process involving cooperative changes in the chain conformation and packing. It is generally considered to be a two-step process involving nucleation and growth. The rate of phase transformation depends mainly on temperature, polymer purity, mechanical stimuli, additives, and other factors.^7–12^ Research has shown that nucleation occurs most rapidly at low temperatures, with an optimum around – 10 °C, while growth is fastest at higher temperatures, with a maximum around 40 °C.^9,13–17^ At the same time, it has been shown that external stimuli such as tensile loading, shear stress, or high-pressure treatment can significantly modify crystallization behavior and promote the formation of specific polymorphic forms.^18–25^

In order to tailor the crystallization kinetics of PB-1, considerable attention was paid to the use of nucleating agents (NA). These provide heterogeneous nucleation centers and are a widely used strategy in polyolefin modification as they accelerate crystallization and improve the homogeneity of properties. Furthermore, from a processing viewpoint, they are a simple and practical approach. In the case of PB-1, it has been shown that various inorganic additives, such as halloysite nanotubes or palygorskite, promote the transformation of form II to form I through a lattice matching mechanism.^26,27^ Similarly, blending with isotactic polypropylene (PP) or the application of β-nucleation systems has been used to modify crystalline stability and increase heat resistance.^15,28^ Despite these advances, however, the influence of organic NAs—especially sorbitol derivatives—has been only limitedly investigated. Among organic NAs, the sorbitol derivative Millad 3988 (1,3:2,4-bis­(3,4-dimethylbenzylidene) sorbitol) has found wide application in PP, where it improves optical clarity by forming nanofibrous structures that act as effective nucleation centers.^29,30^ In PP, this mechanism leads to an increase in the crystallization temperature (*T* *_c_*), accelerated crystallization, and improved transparency and toughness. However, the performance of the agent is limited by its poor solubility in the polymer melt: when the saturation concentration is exceeded, Millad 3988 precipitates and forms a separate phase, which can reduce its nucleation efficiency.^31,32^ Given the structural similarity and partial miscibility of PB-1 with PP, it can be expected that commonly available NAs intended for PP will also be suitable for PB-1.^33^ Similarly, Millad 3988 could be one of them. However, its precise effect on polymorphic transformations, crystallization rate, and the resulting properties of PB-1 have not yet been systematically evaluated.

This work focuses on the completion of the aforementioned knowledge gap by examining in detail the effect of Millad 3988 on the crystallization and phase transitions of the PB-1 homopolymer and its random copolymer. The nucleating agent was incorporated into the polymer matrix at concentrations of 0.2 and 0.6 wt % using twin-screw extrusion to ensure its homogeneous dispersion. The effect was evaluated by using spectrophotometry, wide-angle X-ray scattering (WAXS), and differential scanning calorimetry (DSC). The results obtained provide new insights into the ability of sorbitol nucleating agents to modify PB-1 polymorphism, which is of direct relevance to industrial processing and practical applications.

## 2 Experimental Section

### 2.1 Materials

Two polymeric materials were used in this work. The first was polybut-1-ene homopolymer Koattro PB 0300 M with a melt flow rate of 4 g/10 min (190 °C, 2.16 kg) according to ISO 1133–1. The second was random copolymer PB-1 with a low content of ethylene Toppyl PB 8640 M with a melt flow rate of 1 g/10 min under the same conditions as the previous one. Both polymers were supplied by LyondellBasell Industries.

Nucleating agent Millad 3988 (1,3:2,4-bis­(3,4-dimethylbenzylidene) sorbitol), supplied by Milliken Chemical, was applied in the concentration of 0.2 and 0.6 wt %. This nucleating agent is commonly used as a clarifying agent in polypropylene. Nomenclature of materials in this paper follows this pattern: material Koattro PB 0300 M is denoted as PB-1 and material Toppyl PB 8640 M coPB-1. These are followed by suffix “-0.2N” or “-0.6N” indicating the amount of NA added.

### 2.2 Samples preparation

In the preparation of samples with nucleating agent, paraffin oil was first manually mixed into the PB-1 granules at a concentration of 0.3 wt % for better dispersing of the nucleating agent. Then, the nucleating agent was manually incorporated at concentrations of 0.2 and 0.6 wt %. Subsequently, the mixtures were processed on a twin-screw extruder from LabTech Engineering Co. at a screw speed of 45 ± 5 rpm and the temperature of the heating elements ranged from 180 to 220 °C. The extruded strings were chopped into roller-shaped particles using a granulation machine from Brabender.

For haze, X-ray diffraction, and microscopy measurements, plates with dimensions of 125 × 125 × 0.5 mm were used. Plates were pressed at 210 °C for 5 min using a manual press. Polyethylene terephthalate separation films were placed between the plates and the frame. Subsequent cooling of the plates was carried out at 25 °C for 10 min. The samples were then analyzed at 0, 2, 4, 24, 48, 72, 144, 312, 480, 816 and 1 152 h after cooling.

### 2.3 Methods

The phase composition of the materials and crystallinity were monitored by wide-angle X-ray scattering (WAXS). The XRDynamic 500 diffractometer, Anton Paar, was used, which employs Bragg-Bretano geometry (CuKα radiation and Ni filter). The examined sample was measured with a monochromatic beam with a wavelength of λ=0.154 nm, over a range of diffraction angles of 2θ=5–30° in reflection mode. The total crystallinity of the samples was calculated from eq 1:

$$X_{c}=\frac{I_{c}}{I_{c}+I_{a}}\cdot 100\%$$ 1

where *X* *_c_* is crystallinity [%], *I* *_c_* is area of the crystalline phase (below the diffraction peaks) and *I* *_a_* is area of the amorphous phase (amorphous halo).

The polymorphic composition, or representation of form I and II, was calculated from the individual areas under the diffraction peaks using eq 2:^34^

$$X_{I}=\frac{I_{1}}{I_{1}+0.67I_{2}}$$ 2

where *X* *_1_* is percentage of crystalline form I [%]; *I* *_1_* is area of crystalline form I (below diffraction peak at angle 9.9°) and *I* *_2_* is area of crystalline form II (below diffraction peak at angle 11.9°). A correlation parameter 0.67 considering structure factors proposed by Men^34^ is introduced.

A HunterLab UltraScan Pro spectrophotometer with an optical resolution of 5 nm was used to assess the sample haze. In total, the sample was measured five times at different locations, and the arithmetic mean of the haze value was calculated. Haze was measured according to an ASTM D1003.

Images of the structure were taken by using an Olympus BX41 polarizing optical microscope equipped with an Infinity 2 digital camera. Samples with a thickness of 30 μm were cut from the molded plates after the longest aging time (1 152 h).

The resulting structure after transformation (1 152 h of aging) was also monitored by scanning electron microscopy (SEM). The plates were fractured in liquid nitrogen, and the fracture surface was etched to remove amorphous phases using 1 wt % solution of KMnO_4_ in 85% H_3_PO_4_. Samples were first washed in ethanol, then etched for 15 min, washed in running water, exposed to H_2_O_2_ for 5 min, and then washed again under running water. Samples were then sputter-coated with a Pd/Au alloy. A Phenom Pro SEM (ThermoFisher Scientific) was used for the observation at an acceleration voltage of 10 kV in the backscattered electron mode.

The thermal properties of the materials were characterized using DSC from Mettler Toledo. A sample weighing approximately 5 mg was placed in an aluminum pan. The measurements were carried out in a nitrogen atmosphere at a flow rate of 20 mL/min. The temperature regime of the nonisothermal analysis was as follows: Tempering at 15 °C for 2 min to equilibrate the temperature, heating from 15 to 200 °C at a rate of 10 °C/min, holding for 5 min to ensure the erasing of thermal history, and then cooling to 15 °C at a rate of 10 °C/min. In addition, to verify the efficiency of the nucleating agent as a function of the melt temperature, the same scans were performed at different maximum temperatures in the range 195 to 230 °C. Isothermal crystallization was performed at these conditions: Tempering at 15 °C for 2 min to equilibrate the temperature, heating from 15 to 210 °C at a rate of 10 °C/min, holding for 5 min to ensure the erasing of thermal history, rapid cooling to *T* *_c_* at a rate of 50 °C/min, holding until the crystallization peak is completed. Crystallization temperatures were different for both materials, for PB-1:92, 95, 98, 100, 102, and 105 °C; for coPB-1:72, 75, 78, 80, 82, and 85 °C, respectively.

## 3 Results and discussion

### 3.1 Morphology

The evolution of the morphology of the prepared samples during the time was analyzed by WAXS (Figure 1). Characteristic diffraction peaks for form I and II occur for all analyzed samples. The form I diffraction peaks are located at 9.9°, 17.3°, 20.2° and 20.6°, while the form II diffraction peaks can be identified at 11.9°, 16.9° and 18.5°.

**Figure 1:**
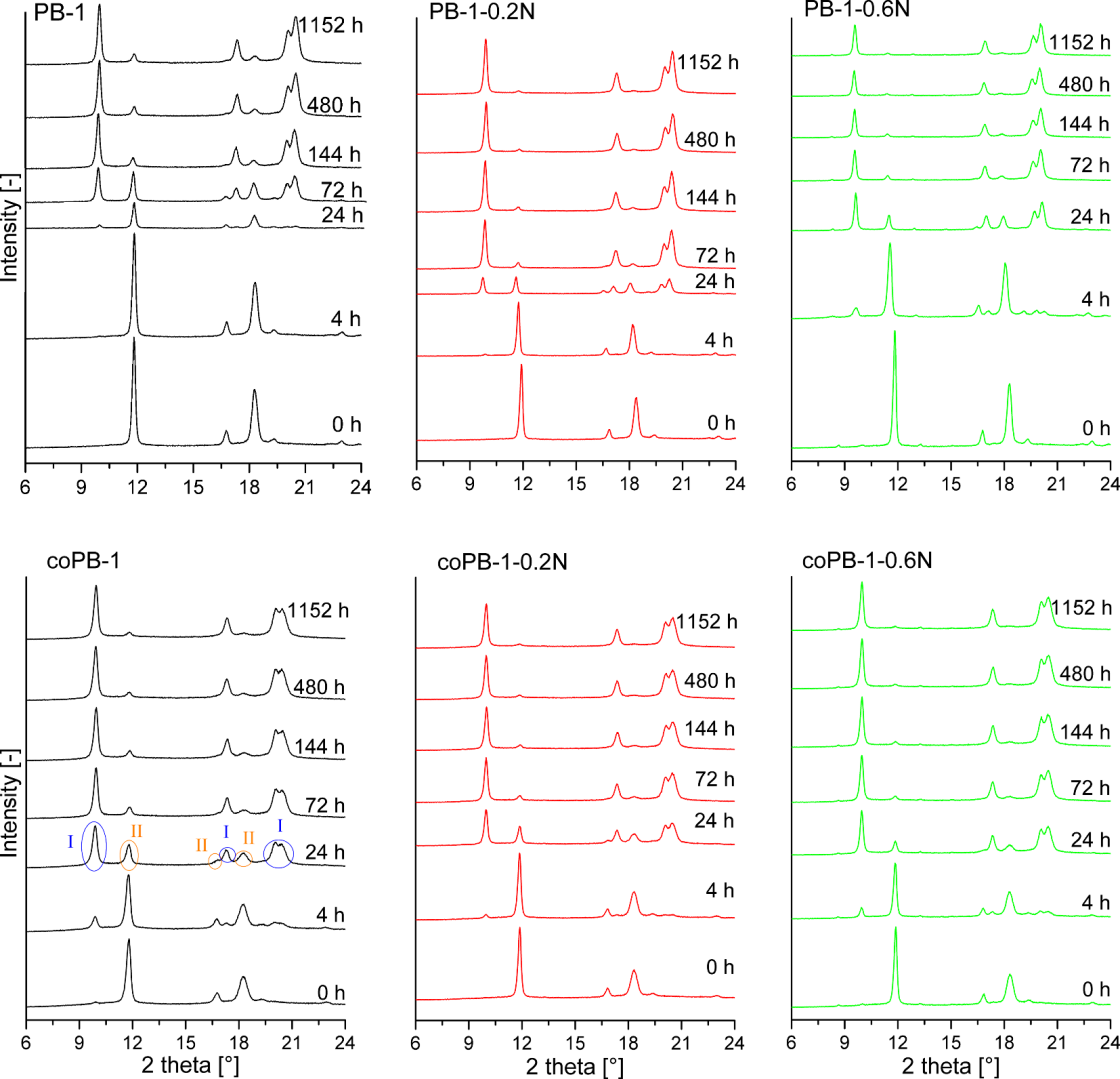
X-ray diffraction patterns of all samples in time.

From diffractograms, the values of crystallinity and percentages of forms I and II in each sample at specific aging times were calculated. Figure 2 graphically shows the crystallinity and form I portion for the measured samples as they age. The crystallinity reaches higher values in the case of the homopolymer (approximately 10% higher) as compared to copolymer, which is expected due to the higher regularity in the chain. In all cases, a slightly higher crystallinity can be observed for the nucleated samples. For all samples, at short aging times, the crystallinity gradually increases, especially in the case of the neat PB-1 homopolymer. Finally, at longer aging times, the crystallinity value stabilizes.

**Figure 2:**
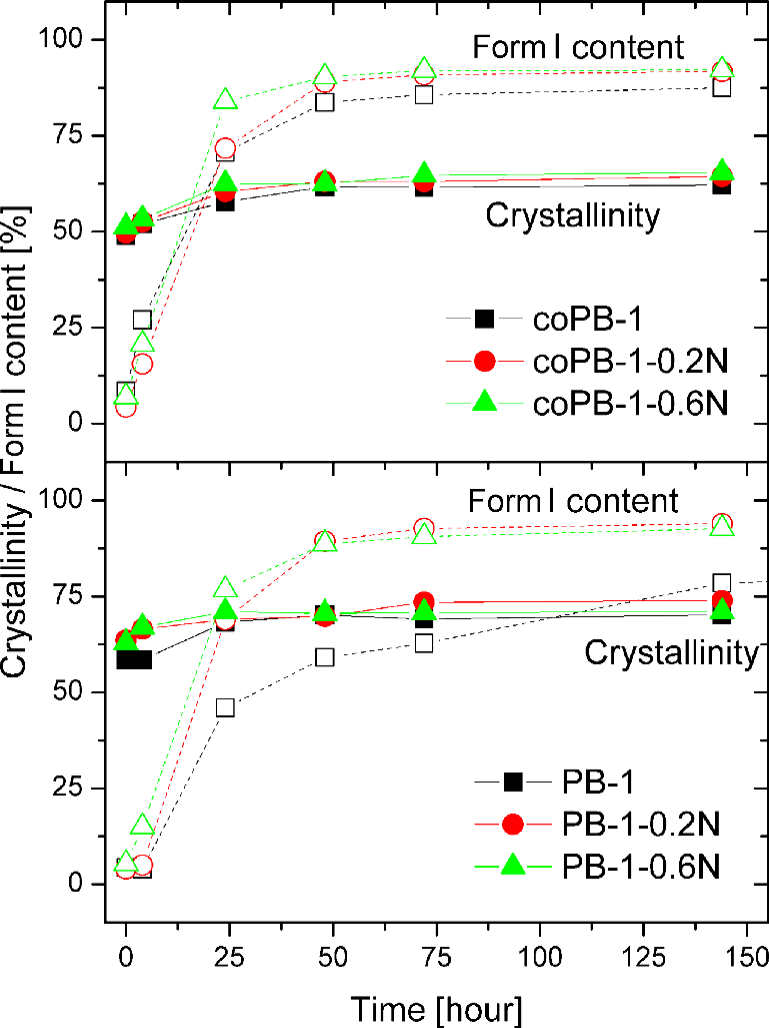
Evolution of crystallinity and form I content of all samples (solid lines represent crystallinity, and dashed lines represent form I content).

The curves of phase transition evolution from form II to form I are also shown in Figure 2 (and Figure S1). The most extensive phase transition occurs between 4 and 48 h of aging, except for the neat homopolymer PB-1. In the case of the homopolymer with the addition of NA, it is clearly seen that a faster phase transition occurs to a final value of about 95% of form I. Neat PB-1 transforms more slowly and even after 480 h of aging still contains about 15% of form II. In the case of the copolymer, the addition of NA does not significantly accelerate the phase transition but leads to a higher percentage of phase transition, about 2% at the end of aging (1 152 h).

A trend very similar to that observed in the evolution of morphology is also evident in the development of haze, as confirmed by the time-dependent haze measurements presented in Figure 3 (and Figure S2). These results show that haze increases shortly after compression molding, reaches a maximum, and subsequently decreases and stabilizes over time (up to 1 152 h), mirroring the crystallization and phase transition dynamics of PB-1 and its copolymer.

**Figure 3:**
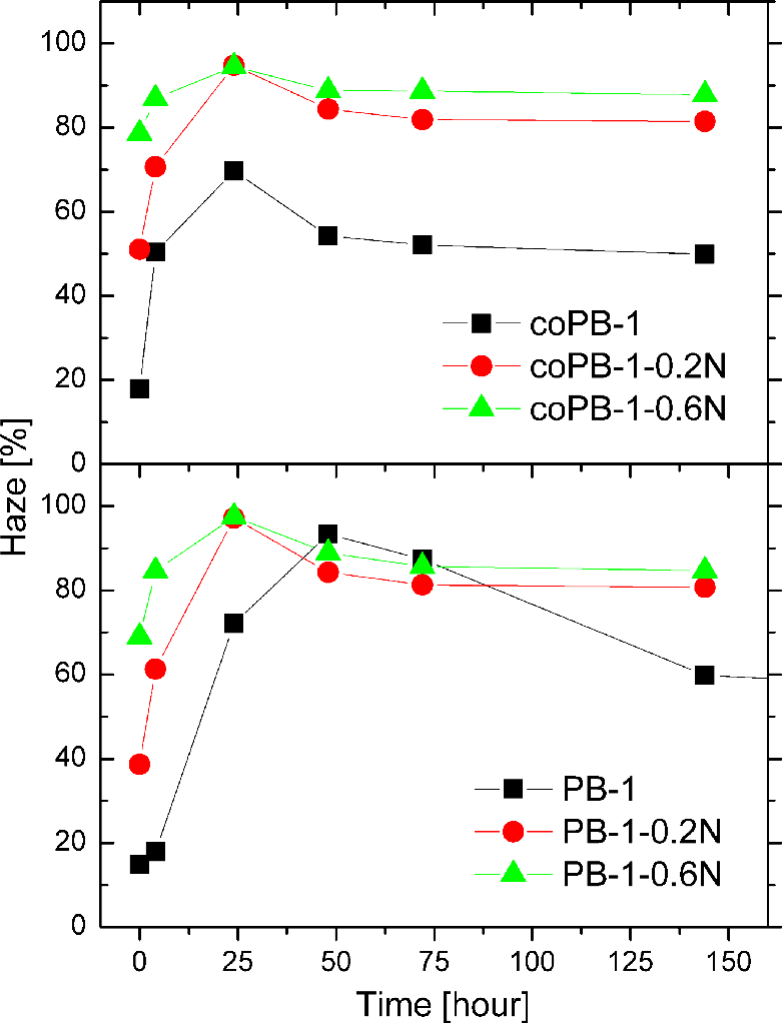
Evolution of haze upon time

Unlike in polypropylene, where NA functions effectively as a clarifier by reducing optical haze, its influence in PB-1 and its copolymer is notably different. Instead of enhancing clarity, the addition of NA leads to a substantial increase in haze, suggesting that in these materials, the agent serves purely as a nucleating additive without clarifying properties.

Across all tested samples, an initial increase in the haze is observed, followed by a decline and eventual stabilization. For homopolymer PB-1, this stabilization occurs significantly earlier in the presence of NA – approximately after 72 h – while the unmodified material reaches a stable haze level only after around 480 h, accompanied by a marginal decline thereafter.

In the copolymer samples, haze levels plateau more rapidly, around 48 and 72 h for samples containing 0.6 and 0.2 wt % of NA, respectively, and approximately 144 h for the neat material. The initial rise in haze is attributed to progressive crystallization into the kinetically favored form II (increase of crystallinity in Figure 2). Concurrently, the onset of the phase transition into the thermodynamically stable form I likely begins. After reaching maximum crystallinity (24 h, and in case of PB-1 48 h), haze decreases due to ongoing transformation, ultimately stabilizing once the transformation to form I is largely complete. Form I exhibits lower haze than form II, which is caused by the reduction in crystal size during phase transformation, as described elsewhere.^5^

These findings underscore the role of the nucleating agent in accelerating the phase transition process, particularly in the homopolymer, as evidenced by the shortened time to haze stabilization. However, the final haze values after stabilization are significantly higher in nucleated samples. In homopolymer PB-1, haze increases from approximately 47% to 79% and 83% with the addition of 0.2 and 0.6 wt % NA, respectively. For the copolymer, values rise from about 46% to 81% and 87% under the same conditions.

Evidently, the nucleating agent fails to induce the formation of an organogel or ultrafine crystallites in PB-1, which would otherwise lead to enhanced clarity, as is typical in polypropylene. Instead, the system behaves as a heterogeneous, haze-promoting composite. While not acting as a clarifier, the NA clearly functions as a heterogeneous nucleating site, particularly in the homopolymer, where it significantly shortens the time required to reach maximum haze – indicating a faster crystallization process overall.

The nucleation effect of the agent used can be easily verified by observing the morphology using a light polarizing microscope. Figure 4 clearly shows that the addition of the nucleating agent leads to the formation of a fine-grained structure – that means a significant reduction of crystallite size in both materials, homopolymer and copolymer.

**Figure 4:**
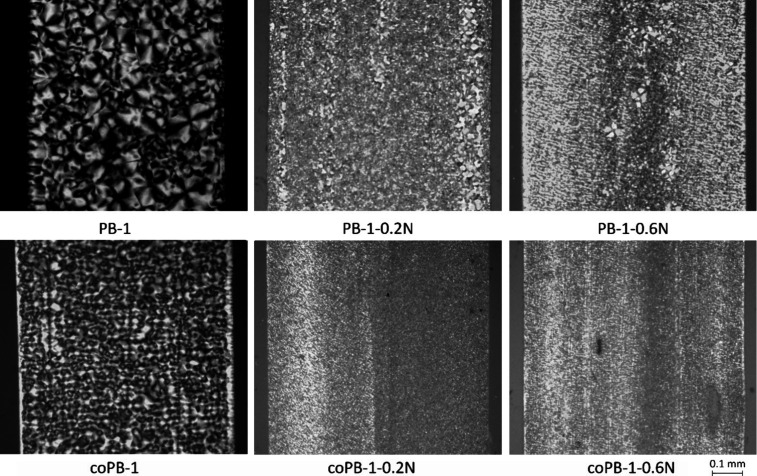
Morphology of the samples

Detailed morphology of compression-molded samples after whole period of aging (after 1 150 h) was observed in scanning electron microscope. The images taken are shown in Figure 5. The images of the samples containing the nucleating agent show holes that are not present in the pure homopolymer and copolymer. The size (diameter) of these holes usually corresponds to the diameter of the rod-shaped crystals (approximately 1 μm) of the NA itself (see Figure S3), except for the homopolymer with 0.2 wt % NA. It is therefore likely that these are traces of NA crystals that have etched off with the amorphous phase. This implies that NA does not dissolve in the polymer melt under the given conditions in copolymer and in homopolymer with higher concentration of NA, instead creating two phase system indeed manifesting higher haze.

**Figure 5:**
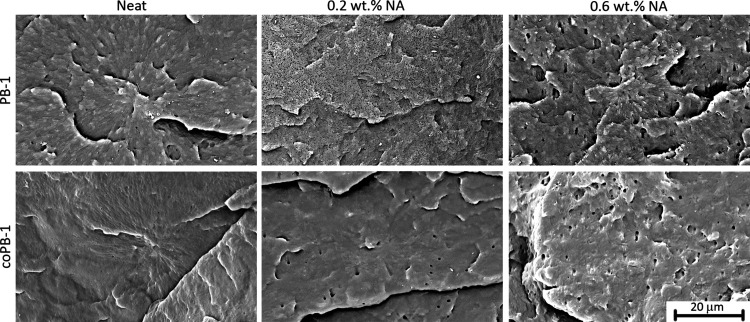
Micrographs of the structure of all samples.

On the contrary, the homopolymer PB-1 with lower NA concentration shows traces of NA of significantly smaller diameter. This would be consistent with dissolution of NA in the melt and subsequent phase transition to form smaller crystals, inducing heterogeneous nucleation of polymer and formation of finer crystal structure.

### 3.2 Thermal behavior

#### 3.2.1 Nonisothermal crystallization

Figure 6 shows the crystallization curves of all measured samples, and Table 1 gives the data from these curves, namely, *T* *_c_* (crystallization peak maximum), heat of crystallization (*ΔH* *_c_*), and width of crystallization peak. It can be seen that *T* *_c_* is significantly lower for the copolymer samples compared to the homopolymer ones. The copolymer without the addition of a nucleating agent (coPB-1) has the lowest *T* *_c_* of 38.6 °C. At the same time, this copolymer also has the largest crystallization peak width, which corresponds to the slowest crystallization. The addition of NA leads to both an increase in *T* *_c_* (by more than 20 °C) and a significant narrowing of the crystallization peak, with a lower concentration of 0.2 wt % being a sufficient amount. Higher concentration of 0.6 wt % do not further accelerate crystallization in copolymer. In the case of the homopolymer, the addition of NA also leads to an increase in *T* *_c_* and a narrowing of the peak, but the changes are not as pronounced. All these results confirm that the applied NA has an effect on the crystallization process of both materials, but it is more pronounced in the copolymer. It can be seen from the Table 1 that the heat of crystallization is higher in the case of the homopolymer, which corresponds to a higher crystallinity.

**Figure 6:**
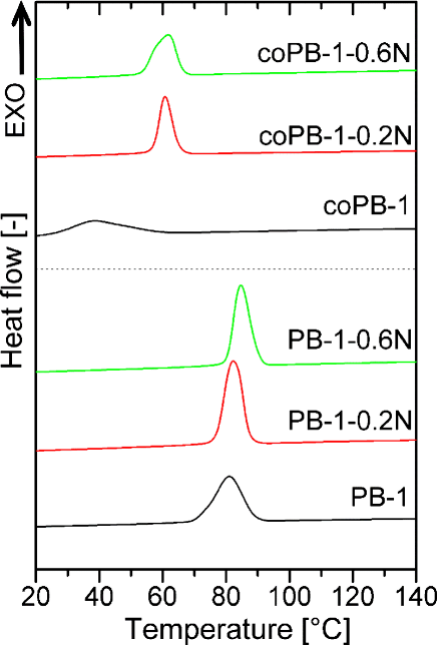
Crystallization curves of all samples.

**Table 1:** Values of *T*_c_, ΔH_c_ and width of crystallization peak obtained from DSC evaluation for each sample

Material	*T*_c_[°C]	ΔH**_c_** [J/g]	Width of crystallization peak [°C]
**PB-1**	81.20	42	17.06
**PB-1–0.2N**	82.62	41	10.31
**PB-1–0.6N**	84.92	40	10.02
**coPB-1**	38.60	24	33.65
**coPB-1–0.2N**	61.01	25	8.07
**coPB-1–0.6N**	62.01	26	12.73

The effect of the sorbitol nucleating agent used in polypropylene is given by its solubility in the polypropylene melt, which is affected by the final melting temperature.^32^ However, the solubility of NA in PB-1 under the given sample preparation conditions was not confirmed, as shown by the haze increase (Figure 3). For this reason, nonisothermal crystallization of PB-1 was also performed after previous melting at different temperatures, namely 200 to 230 °C. The corresponding crystallization curves are shown in Figures 7 and 8. The effect of melting temperature on subsequent crystallization was observed only in the case of a low NA concentration, especially in the copolymer. Shift in *T* *_c_* to lower values with increasing temperature of melting indicate the solubility, or partial solubility at least, of the nucleating agent at such low concentration in the melt of PB-1. In this case, upon cooling, NA crystallizes first and then polybut-1-ene and crystallization may be slowed down. The crystallization of coPB-1–0.2N has been studied in more detail – additional scans have been taken at other temperatures of melting. The results are presented in Figure S4. The shift of *T* *_c_* to lower values occurs gradually between temperatures of melting between 205 and 220 °C, where presumably the dissolution of NA in the melt occurs.

**Figure 7:**
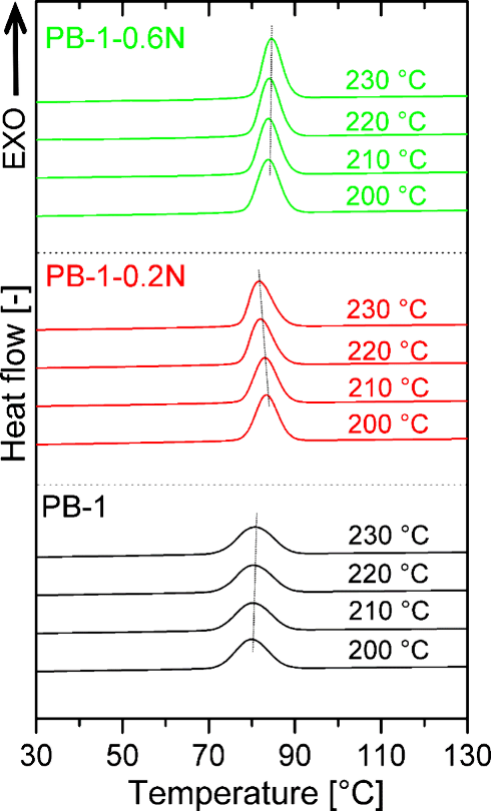
Crystallization curves of PB-1 homopolymer samples after melting at different temperatures

**Figure 8:**
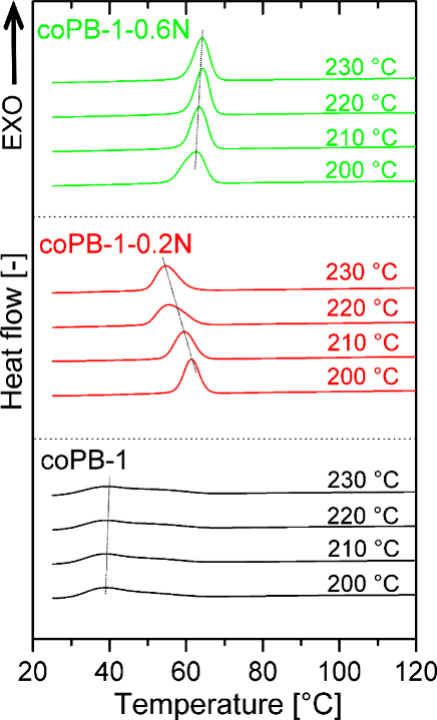
Crystallization curves of PB-1 copolymer samples after melting at different temperatures

After controlled crystallization (Figure 6) the samples were melted and from the melting endotherms (see Figure S5), melting temperatures and heat of melting values for form I and form II over time since compression molding were obtained (Figures 9 and 10). The results show a gradual transformation from form II to form I. At time 0 h, all samples show only one melting peak at about 116 °C for the homopolymer and about 100 °C for the copolymer, corresponding to the melting of form II. Gradually over time, a peak appears and grows at about 130 and 115 °C, respectively, corresponding to the melting of form I. The largest change occurs between times 4 and 24 h (Figure 9). Form I was detectable in the homopolymer after only 2 h of aging and its temperature increased slightly with time. The values are very similar for pure and nucleated samples. In contrary, the melting temperature of form II decreased slightly over time due to transformation into form I, finally it was not detectable by DSC at all – at 144 h for nucleated samples and slightly later at 312 h for pure PB-1. This suggests that the presence of the nucleating agent accelerates the phase transition process for the PB-1 homopolymer. In all copolymer samples, form I was detectable after 2 h, with the melting temperature of this form increasing slightly with time. The increase in melting temperature is associated with the increase in lamella thickness and refinement of crystallites. The melting temperature of the pure copolymer is significantly lower than that of the nucleated samples, by about 6 °C. This drop in melting temperature is linked with wider crystal size distribution, which is manifested by a broad crystallization peak; see Figure 6. It is therefore evident that crystallites formed in the presence of NA are more perfect – however, these are crystallites formed by transformation from form II. The melting point of form II is similar for all samples. It also appears that the presence of NA accelerates the phase transition process in this material as well, since the melting peak of form II is only detectable up to an aging time of 4 h for the nucleated samples, whereas it is 24 h for the pure copolymer.

**Figure 9:**
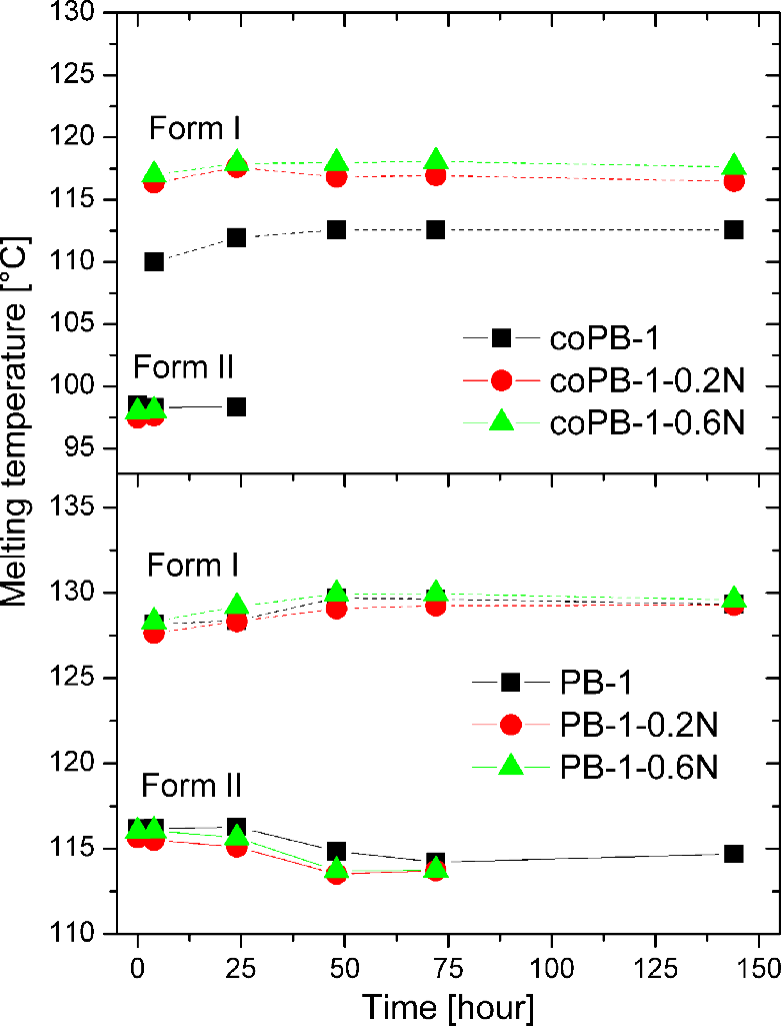
Evolution of melting temperatures of forms I and II of all samples upon aging.

**Figure 10:**
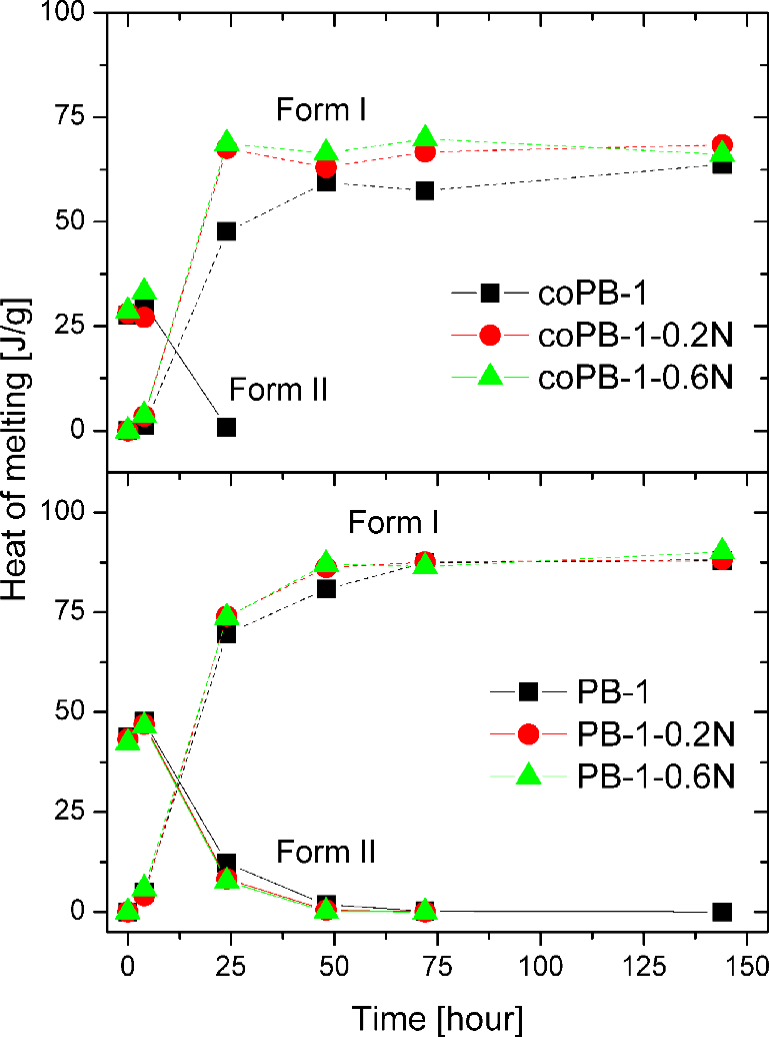
Evolution of Heat of Melting of Forms I and II of all samples upon aging.

The evolution of the heat of melting over time for all samples can be seen in Figure 10. At short times (up to 4 h), an increase in the values can be observed for form II, which is due to gradual crystallization. This is followed by a sharp decrease to almost 0 at 24 h for the copolymer and 48 h for the homopolymer. There is a further gradual decrease to 0 as a result of transformation into form I. On the other hand, the evolution of the melting heat of form I has an increasing trend with a S-shape profile. At first, the values increase slightly up to an aging time of 4 h, then there is a sharp increase and then a plateau with a slight increase. In the case of the homopolymer, the effect of NA is not very noticeable; however, a significantly slower transformation and lower heat of melting values for form I can be observed for the copolymer. Thus, it is proven that the nucleating agent influences the course of the phase transition.

#### 3.2.2 Isothermal crystallization

Isothermal crystallization of the PB-1 homopolymer was carried out at 92, 95, 98, 100, 102, and 105 °C. The obtained crystallization exotherms of neat and nucleated samples are shown in Figure S6 and corresponding crystallization half-times (*t1/2*) in Figure 11 and Table 2. At lower crystallization temperatures (92 to 98 °C), the NA obviously performs its function and slightly accelerates the crystallization process. There is no significant difference in efficiency between samples with different concentrations of the nucleating agent. However, as the temperature increases, the exotherm of the sample containing 0.2 wt % NA starts to approach that of the neat homopolymer, even at *T* *_c_* 100 °C, it has a longer crystallization half-time than the neat polymer. It can therefore be seen that NA used in lower concentrations at high crystallization temperatures no longer works. This may be due to the solubility of NA in the PB-1 melt and its lack of crystallization from solution at high crystallization temperatures. This may then lead to dilution of the system and slow the crystallization of PB-1.

**Table 2:** Values of crystallization half-times

***T*_c_ [°C]**	**Crystallization half-time [min]**		
	**PB-1**	**PB-1–0.2N**	**PB-1–0.6N**
**92**	1.97	1.38	1.13
**95**	3.17	2.27	2.35
**98**	5.57	5.10	4.52
**100**	11.78	12.43	8.73
**102**	13.43	14.05	10.05
**105**	34.97	47.90	23.23
***T***_**c**_ **[°C]**	**coPB-1**	**coPB-1–0.2N**	**coPB-1–0.6N**
**72**	4.30	1.25	1.12
**75**	8.85	2.72	2.58
**78**	11.33	4.72	4.02
**80**	31.78	8.10	7.22
**82**	43.35	13.85	15.05
**85**	-	30.17	28.93

**Figure 11:**
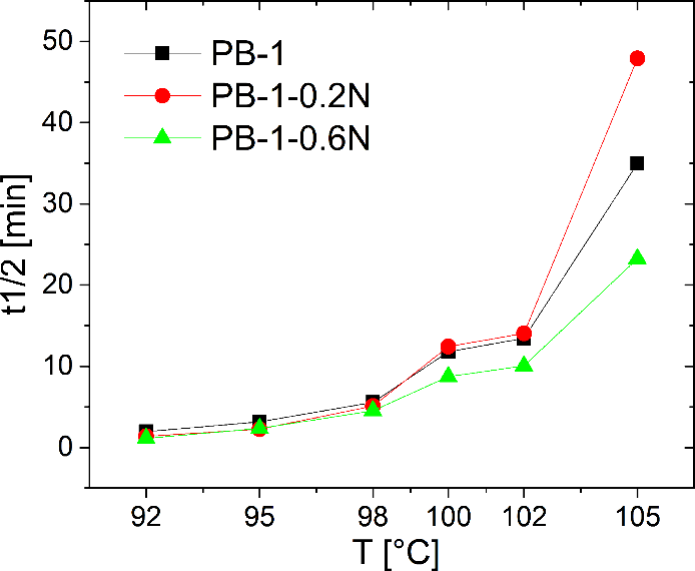
Crystallization half-times of isothermally crystallized homopolymers

Isothermal crystallization of the PB-1 copolymer was carried out at 72, 75, 78, 80, 82, and 85 °C. The obtained crystallization exotherms of neat and nucleated samples are shown in Figure S7 and corresponding *t1/2* in Figure 12 and Table 2. The results show that the NA affects the crystallization kinetics of the PB-1 copolymer. However, the effect of NA concentration does not play a significant role upon stated conditions contrary to PB-1 homopolymer, see Figure 11. In fact, the applied crystallization temperatures for both types of PB-1 are not the same. Apparently, the crystallization temperatures for the copolymer are low enough for the crystallization of dissolved NA in the melt of the PB-1 copolymer before its own crystallization. Thus, even a small amount of 0.2 wt % significantly accelerates the crystallization process; see Table 2. It can therefore be confirmed that the commercial sorbitol-based nucleating agent Millad 3988, originally designed for polypropylene, is also effective in the case of PB-1 copolymer.

**Figure 12:**
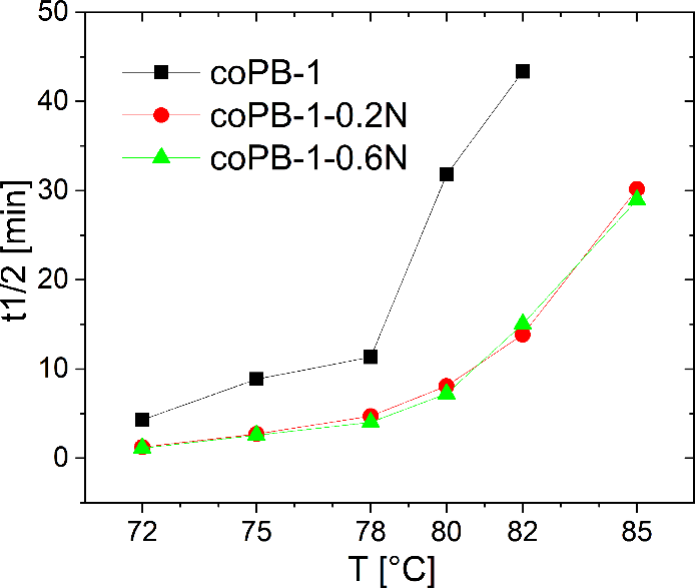
Crystallization half-times of isothermally crystallized copolymers.

As mentioned above in the text, in the case of polypropylene, this nucleating agent is soluble in its melt and on cooling crystallizes first to form fibrils – forming a dense network with a large surface area – and then PP crystallites begin to grow on the surface of the fibrils. Because of the large nucleation surface area and thus the presence of many nucleation sites, these are very small, smaller than the wavelength of light, and thus act as a transparency-enhancing clarifying agent in PP.^32^ However, in the case of the PB-1, the mechanism of action is likely to be different, and the solubility in the melt is contingent by temperature of melting and NA concentration. As suggested by results in this study no nanofibrous structures was observed in the PB-1 matrix.

Comparing the results from nonisothermal and isothermal crystallization and microscopy, it can be concluded that NA Millad 3988 is soluble at low concentration in the melt of PB-1 homopolymer and copolymer at higher melting temperatures, while it dissolves better in the homopolymer. At the melting temperature of 220 °C, it appears that NA is completely soluble in PB-1 melt (shift of the crystallization peak in nonisothermal crystallization), however even at lower temperatures around 210 °C (used in this work), the nucleating agent is at least partially dissolved, especially in homopolymer (SEM image of the structure with small ″holes″ after NA, Figure 5, loss of efficiency at high crystallization temperatures in isothermal crystallization of homopolymer, Figure 11 and S6). For the copolymer, in isothermal crystallization, NA is effective at all crystallization temperatures, but these are considerably lower than in the case of the homopolymer and any dissolved NA, if it dissolved at temperature of melt of 210 °C, would have already transformed.

## 4 Conclusions

In this work, the effect of a commercial sorbitol-based nucleating/clarifying agent, originally designed for polypropylene, on the crystallization and phase transition of polybut-1-ene was investigated. This nucleating agent accelerates the crystallization of both the homopolymer and, in particular, the low-ethylene-content PB-1 copolymer. It increases *T* *_c_* and decreases the crystallization half-time.

However, compared to PP, it does not work as a clarifying agent in PB-1, but instead increases the haze. This may be caused by the limited solubility of NA in the PB-1 melt and its inability to form an organogel. At the higher concentration tested, 0.6 wt %, it does not dissolve in PB-1 melt under the conditions used. At the lower concentration of 0.2 wt % it dissolves, but not unconditionally: the temperature of melting before crystallization is important. At higher temperatures (from about 220 °C) dissolution occurs, which then affects the subsequent crystallization of PB-1. In the case of the PB-1 homopolymer, the addition of NA at a concentration of 0.2 wt % even led to a prolongation of the crystallization half-time compared to pure PB-1 during isothermal crystallization at high temperature. This is not observed for the copolymer due to the significantly lower temperature region of crystallization of polymer than NA.

The transformation from the kinetically preferred form II to the thermodynamically more stable form I over time is also affected by the addition of NA used. An acceleration of the phase transition process was observed for both materials, homopolymer and copolymer, but more significantly for the homopolymer. In both cases, phase transition also occurred to a greater extent after reaching the steady state.

## Supplementary Material



## Notes

Evolution of crystallinity and form I content of all samples upon whole aging period, the evolution of haze upon whole aging period, micrograph of the nucleating agent Millad 3988 before mixing it into the polymer, crystallization curves of coPB-1–0.2N samples after melting at different temperatures, melting curves of all samples in selected times of aging, exotherms of isothermally crystallized PB-1 homopolymer at several *T* *_c_*, exotherms of isothermally crystallized PB-1 copolymer at several *T* *_c_* (PDF). The Supporting Information is available free of charge at https://pubs.acs.org/doi/10.1021/acsomega.5c08056.
