# Supplementary material for: Fast-Track Crystallization of PB‑1 with Sorbitol-Based Nucleating Agent
Source: ACS Omega. 2025 Oct 20;10(42):50521–9. doi: 10.1021/acsomega.5c08056 (PMC12573048; doi:10.1021/acsomega.5c08056)

Supporting Information for Publication

Title

Fast-Track Crystallization of PB-1 with Sorbitol-Based Nucleating Agent

Author List

Jana Navratilova[a], Lenka Gajzlerova[a*], Roman Cermak[a], Martina Polaskova[a,b]

[a]*Department of Polymer Engineering, Faculty of Technology, Tomas Bata University in Zlin, Vavreckova 5669, 760 01 Zlin, Czech Republic*

[b]*The Centre of Polymer Systems, Tomas Bata University in Zlin, trida Tomase Bati 5678, 760 01 Zlin, Czech Republic*

*Email: *lgajzlerova@utb.cz*

Figure S1 Evolution of crystallinity and form I content of all samples upon whole aging period

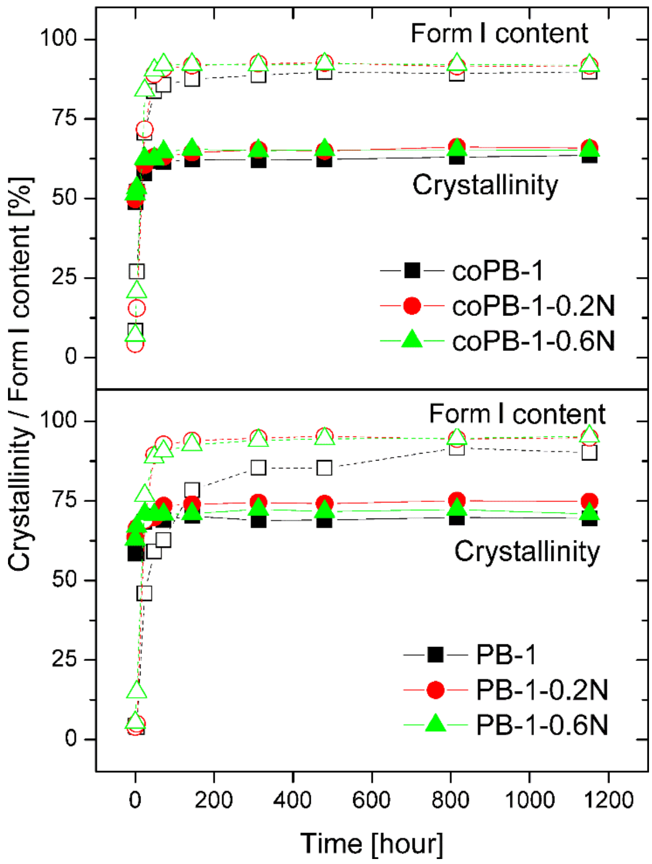

Figure S2 The evolution of haze upon whole aging period

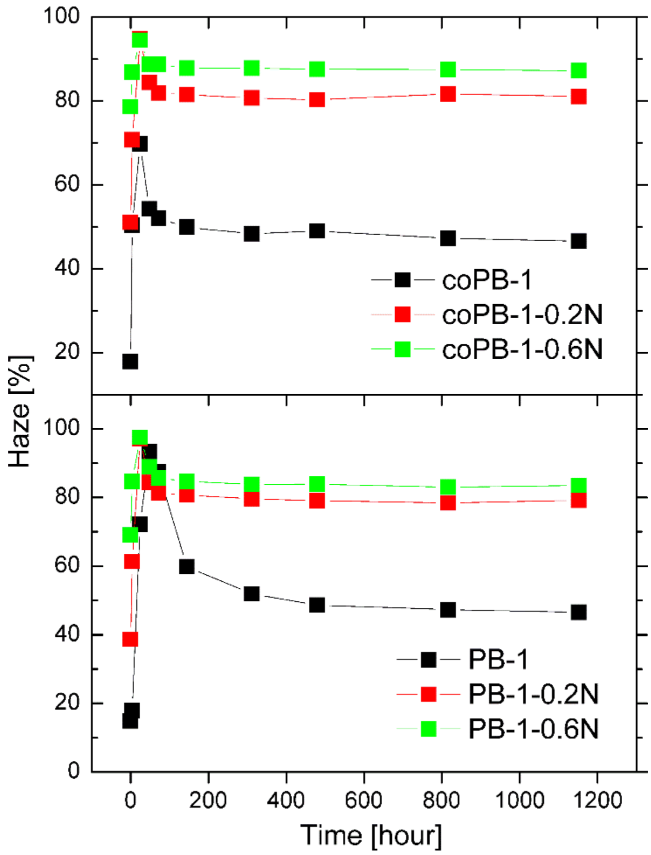

Figure S3 Micrograph of the nucleating agent Millad 3988 before mixing it into the polymer

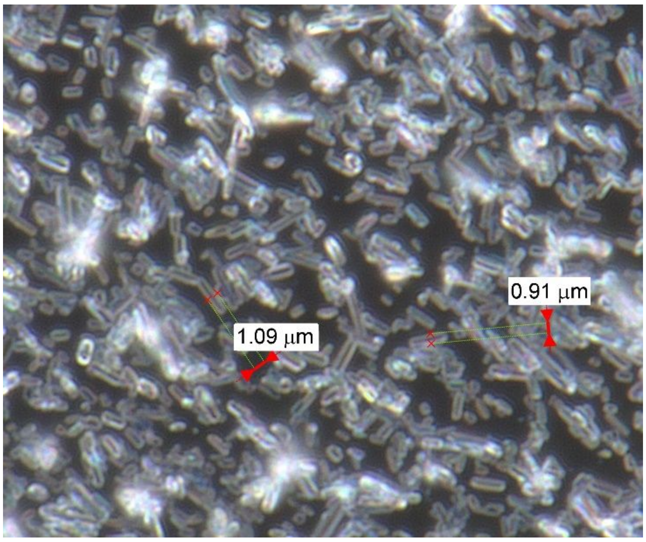

Figure S4 Crystallization curves of coPB-1-0.2N samples after melting at different temperatures

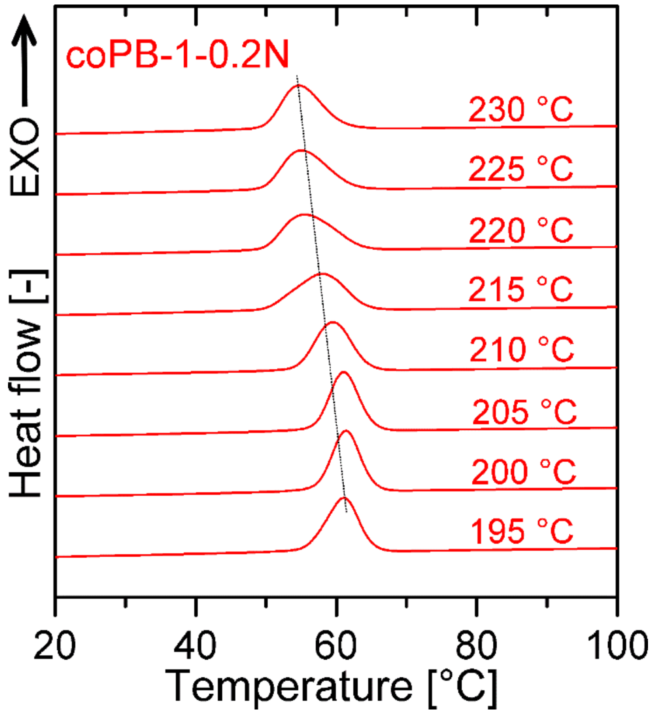

Figure S5 Melting curves of all samples in selected times of aging

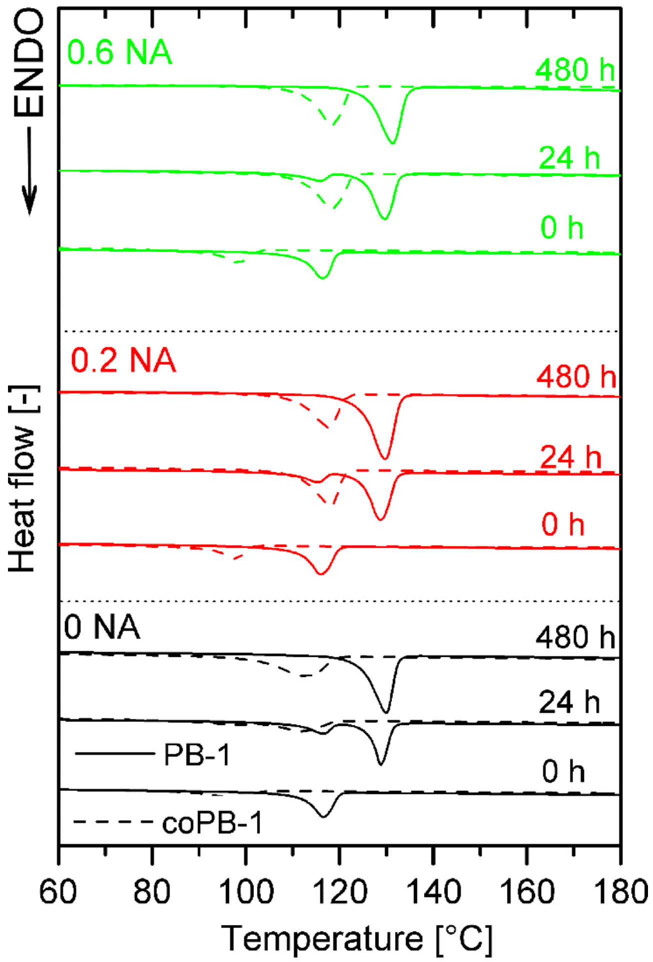

Figure S6 Exotherms of isothermally crystallized PB-1 homopolymer at several T_c

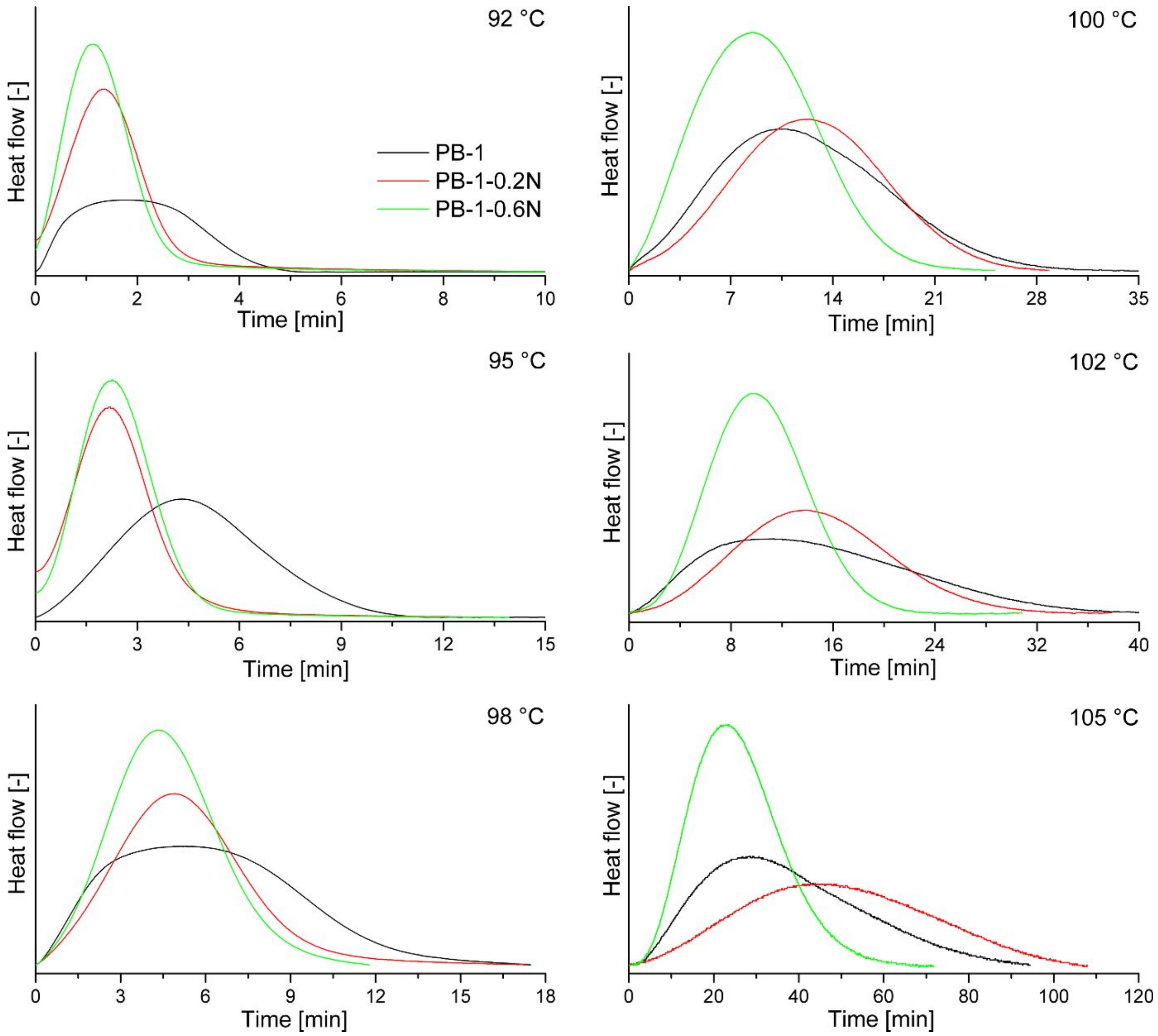

Figure S7 Exotherms of isothermally crystallized PB-1 copolymer at several T_c

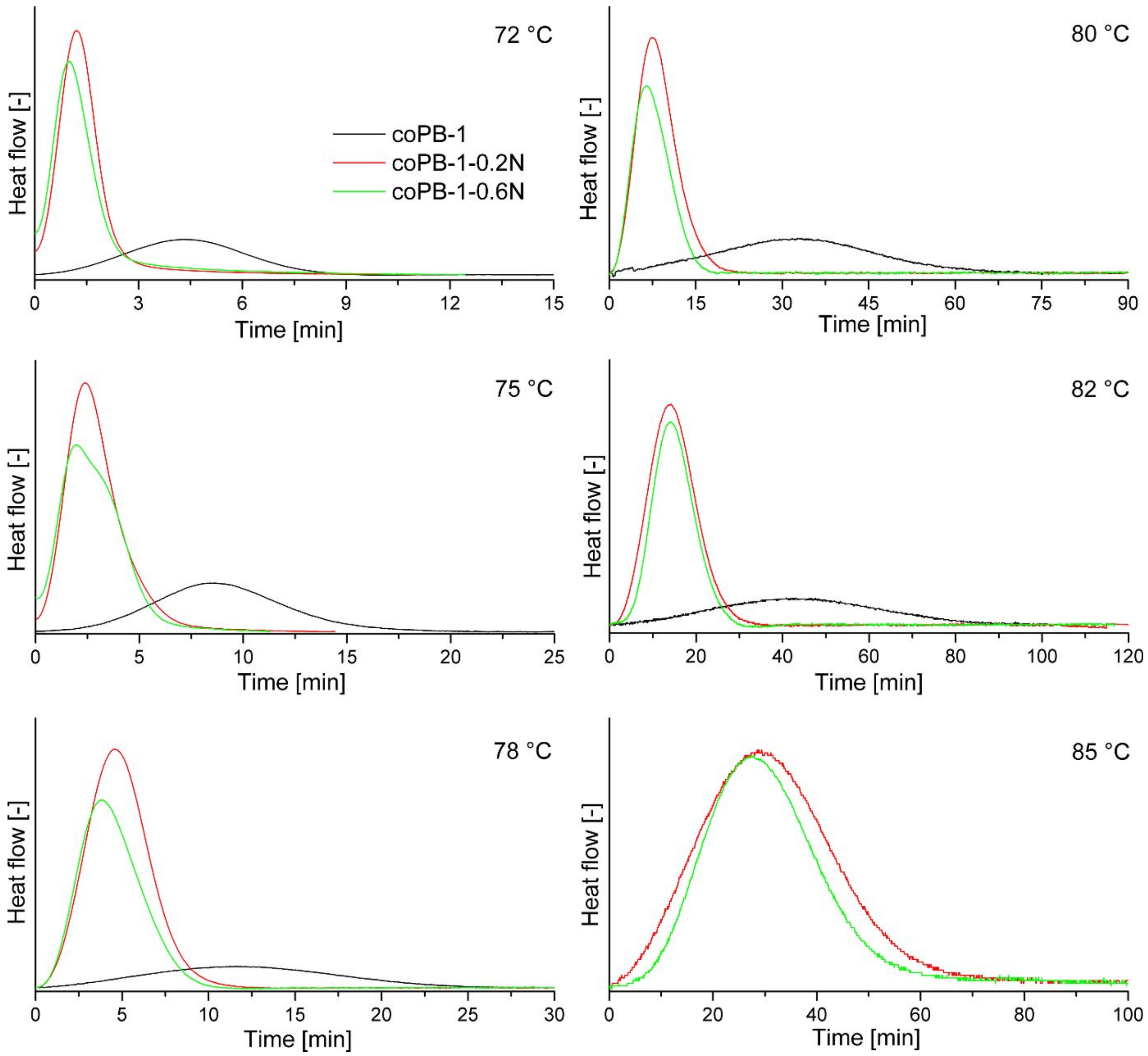

Supplement: Supplementary file 1 [file ao5c08056_si_001.pdf]
